# Supplementary material for: Imeglimin Alleviates High-Glucose-Induced Bioenergetic and Oxidative Stress Thereby Enhancing Intercellular Adhesion in H9c2 Cardiomyoblasts
Source: Int J Mol Sci. 2025 Sep 12;26(18):8913. doi: 10.3390/ijms26188913 (PMC12469297; doi:10.3390/ijms26188913)
Supplement: Supplementary file 1 [file ijms-26-08913-s001.zip › ijms-3833043-supplementary.pdf]

Supplemental Table S1 Sequences of primers of qPCR

		Sequence	Exon Location	RefSeq Number
rat RPLP0	Probe	5'-/56-FAM/CCTGTCTTC/ZEN/CCTGGGCATCACG/3IABkFQ/-3'	1-2	NM_022402
	Primer2	5'-TGTCTGCTCCCACAATGAAG-3'		
	Primer1	5'-CAATCCCTGACGCACCG-3'		
rat Gja1	Probe	5′-/56-FAM/AGTGAAAGA/ZEN/GAGGTGCCCAGACATG/3IABkFQ/-3′	1-2	NM_012567
	Primer2	5′-GGTGGAGTAGGCTTGGAC-3′		
	Primer1	5′-CCTTTGACTTCAGCCTCCAA-3′		
rat Ctnnb1	Probe	5′-/56-FAM/ACGCCCTCC/ZEN/ACGAACTGC/3IABkFQ/-3′	9-10	NM_053357
	Primer2	5′-ACCCTTCAACTATCTCCTCCA-3′		
	Primer1	5′-GTACGAGCACATCAGGACAC-3′		
rat Cdh2	Probe	5′-/56-FAM/TCCCGGCGT/ZEN/TTCATCCATACCAC/3IABkFQ/-3′	14-15	NM_031333
	Primer2	5′-CCTCTCCTCCACCTTCTTCA-3′		
	Primer1	5′-GCTGATCCTTGTCCTCATGT-3′		
